# A Little-Known Form of "Cold Abscess."

**Published:** 1911-01-14

**Authors:** 


### SPOROTRICHOSIS.

### A LITTLE-KNOWN FORM OF "COLD ABSCESS."

Most of the work on this infection has been done by French observers since 1903, when sporotrichosis in man was investigated by De Beurmann and Ramond.

The sporotrichum is a fungus of which there are about seven species, the three best known ones being S. Schenki, S. Dori, and S. de Beurmanni. The species with which we are at present concerned is the S. de Beurmanni, which has been found in South America, where it is common among rats, and in Madagascar.

### The Tumours.

Since 1906 over a hundred cases have been reported, and many investigations made of the disease produced by infection with this sporotrichum. The disease is characterised by the presence of soft swellings under the skin or in the muscles, but also sometimes found in the mucous membranes and the bones. These swellings closely resemble, both histologically and to the naked eye, the "cold abscesses" of tuberculosis and the gummata of syphilis. The fluid in the swellings is a syrupy liquid, clear or streaked with lines of pus, but later becoming thick and opaque. Histologically, the nodules may be said to have three zones, like the granulomata, viz. an outer zone showing perivascular inflammation with round cell infiltration; a middle zone with giant cells and epithelioid cells; and, in the centre, polynuclears and suppurative processes.

### The Essential Cause.

The parasite belongs to the hyphomycetes, and the S. de Beurmanni persents under the microscope a tangled mass of filaments about 2 $\mu$ in diameter, with very numerous spores attached along the filaments or at the extremities of lateral branches. The sporotrichum grows well on glucose agar at an optimum temperature of 22° C., the colonies becoming rapidly pigmented and assuming a chocolate colour, while morphologically resembling cerebral convolutions. The S. de Beurmanni is virulent for the rat, but the S. Dori and the S. Schenki are not virulent, or only slightly so, for this animal.

The commonest form of the disease is the disseminated sporotrichosis of de Beurmann and Gougerot, which appears to be a septicæmia with local lesions determined by traumatism. Another and rarer form may arise from a cutaneous inoculation of the parasite, and produces a lymphangitis spreading from the so-called sporotrichotic "chancre" (E. Brumpt). The gummatous swellings tend to ulcerate, and after healing has occurred a scar is left closely resembling those of tertiary syphilitic lesions.

The presence of the spores in the blood of a case of the generalised disease has been demonstrated by Widal and Weill, and this will explain the action of injury in giving rise to local abscesses in an individual already affected. The blood of infected patients will give an agglutination reaction with an emulsion of the sporotrichum in a dilution of 1 in 500 to 1 in 200, and sometimes even in a 1 in 1,500; it has also been found that the serum of persons infected with actinomycosis, or with the oidium albicans, will agglutinate similarly, but a lower dilution is necessary—up to about 1 in 150.

### An Exceptional Case.

This sporotrichum is an ordinary saprophyte, and has been found growing on certain plants and, by one observer, on oat grains. It gains access to the body by means of a prick or bite, and also through the alimentary canal. Only a few months ago a case was reported in the *Lancet* of a woman in Paris who had been bitten on both thumbs by a rat which had been inoculated with a sporotrichum. Some days afterwards typical indolent circumscribed nodules appeared at the site of the bites, and later a superficial abscess followed, the pus of which had the viscidity characteristic of the lesions of this infection. When the epidermis was removed each nodule was seen to be surmounted by a grey sluggish ulcer. There was no appreciable lymphangitis, but on one side there was some enlargement of the glands, without obvious inflammation. The nature of the infection was soon settled by cultural experiments, and the patient was put on iodine, and the lesions rapidly improved. From the pus pure cultures of the Sporotrichum Jeanselmi were obtained.

### Diagnosis.

With a more general recognition of the value of the methods of parasitological diagnosis it is likely that this infection will be more often suspected clinically. Among the chief points of importance are the maintenance of a general feeling of well-being; the torpidity and multiple character of the lesions; the colour and consistence of the pus; and the almost constant absence of glandular enlargement.

The diagnosis may have to be made from tertiary syphilitic lesions, from the cold abscesses of tuberculosis, from various mycoses, from chronic staphylococcic abscesses, and from fibro-lipomata and sarcomatosis. In the horse and the mule sporotrichosis has been confounded with glanders and with certain forms of lymphangitis.

The prognosis of sporotrichosis infection is exceedingly good, but when it occurs in conjunction with another disease, such as tuberculosis, then it may considerably add to the gravity of the latter. The treatment is very simple, and may be summed up in one word—iodine. A cure should be obtained in a matter of a few weeks by the administration of potassium iodide, or one of the combinations of iodine with albuminoid substances. Large doses of iodide are recommended: German observers have found the best results follow the administration of the salt in combination with iron and arsenic.

